# Femtosecond Laser Fabrication of Micro and Nano-Structures on CIGS/ITO Bilayer Films for Thin-Film Solar Cells

**DOI:** 10.3390/ma14092413

**Published:** 2021-05-06

**Authors:** Huizhu Yang, Gedong Jiang, Wenjun Wang, Xuesong Mei

**Affiliations:** 1 State Key Laboratory for Manufacturing System Engineering, Qujiang Campus, Xi’an Jiaotong University, Xi’an 710054, China; yhzhappy@foxmail.com (H.Y.); gdjiang@mail.xjtu.edu.cn (G.J.); xsmei@mail.xjtu.edu.cn (X.M.); 2 Department of Mechanical Engineering, Faculty of Mechanical Engineering, Xi’an Jiaotong University, Xi’an 710054, China

**Keywords:** bilayer films, ripples, porous structures, micro- and nanostructures

## Abstract

Cu(In, Ga)Se_2_ (CIGS) thin films have attracted considerable interest as potential photovoltaic solar cells. Moreover, several current studies are focusing on improving their conversion efficiency. This study proposes a method to process micro- and nanostructures onto the surface of CIGS/ITO bilayer films to broaden the field of solar cell application. The bilayer films exhibited optical characteristics different from those of a single-film during processing. Field intensities at different layer positions of the CIGS/ITO bilayer films were analyzed, and different structures were fabricated by varying a set of parameters. Ripples were obtained using a pulse energy of 0.15 μJ and scanning speeds in the range of 0.1–1 mm/s, but after increasing speed to 3–5 mm/s, ripple structures were produced that had a large period of several microns and spatial porous nanostructures. This pattern exhibited low reflectivity. Optimal structures were obtained at a scanning speed of 3.5 mm/s a pulse energy of 0.15 μJ, and a reflectivity lower than 5%. Large areas characterized by micron-sized ripple structures and accompanied by nanoscale porous structures presented high optical performance and efficiency, which can be used to broaden the application of thin film-based solar cells.

## 1. Introduction

Cu(In,Ga)Se_2_ (CIGS)-based thin-film solar cells play a pivotal role in the photovoltaic market due to their high efficiency [1,2]. Currently, CIGS products have an efficiency of 10–18% in practical applications [3,4], so research is focused on improving their conversion efficiency to obtain a broader application in industrial production.

CIGS films are direct band-gap semiconductors having an absorption coefficient of up to 10^5^ cm^−1^ and an absorption thickness of 1–2 μm [5]. Currently, most investigations focus on improving the photoelectric conversion efficiency of the CIGS solar cells by adjusting the material composition or optimizing thin-film preparation technology [6,7,8,9]. Different fabrication methods (e.g., co-evaporation, continuous evaporation, sputtering, vacuum evaporation, and spray pyrolysis [10,11,12,13,14]) can modify the elemental composition of the films, thus affecting surface particle size and absorptivity. In addition, heat treatment in different atmospheres can alter the surface morphology of films so that their bandwidth structure and photoelectric properties can be adjusted [2,15]. However, their elemental content changes upon volatilization during heating at high temperature, a process for modifying specific photoelectric properties that is difficult to control. Considering the narrow compositional stability window of a CIGS quaternary compound, the fabrication of surface structures has attracted more attention because of its specific light-trapping advantages. Common techniques for processing surface structures (e.g., nanoimprint lithography and dry etching [16], laser scanning holographic lithography [17], two-beam laser interference aided photoetching [18], and other relevant techniques [19,20,21]), are not ideal due to the limitation of processed materials and complicated processes. In contrast, using a laser to fabricate periodic surface structures is simple: it can process most kinds of material without changing their elemental composition, an advantage that is more obvious in the difficult processing of thin films. The fabrication of surface structures on thin films is controllable, and with an ultrafast laser, the composition and properties of thin films, especially those with special properties, do not change during a cold working process, something that is beneficial for thin-film devices. So far, the majority of current studies have focused on single-layer thin films [22,23], but practical devices are usually made of multiple layers. The influence of another layer is always unavoidable so it must be taken into consideration during processing.

As the study of a single-layer film in our previous research [24,25] showed, there are some problems with the laser processing of high-transmittance thin films. When the laser irradiates the surface of ITO thin films (a typical high-transmittance material), most of the energy passes through the film and is deposited at the interface of the ITO thin film and its glass substrate. In addition to the surface’s periodic structure, the film is ablated and damaged. Based on this, the light-absorbing material in a CIGS thin film is suited for use in the lower layer, so that when the energy passes through the upper layer of a high transmittance thin film it is transferred to the lower layer instead of to the glass substrate. The two typical materials of ITO and CIGS thin films were selected because the ITO thin film has similar light-transmission characteristics to other window layers and to the buffer layer in a CIGS thin-film solar cell.

Based on this, a method of using an ultrafast laser to modify the surface morphology of CIGS/ITO bilayer thin films is proposed, and the interaction between the laser and the different properties of the thin films (high-transmittance and light-absorption materials) is studied. Bilayer films exhibit different optical characteristics compared to single-layer films [26] because the light intensity distribution changes due to mutual interference. Therefore, structures can be fabricated onto the surface of bilayer films and modified by adjusting the processing parameters, whereas the optical properties of the films can be varied by using different surface structures. These composite structures can expand the field of CIGS-based solar cell application.

## 2. Materials and Methods

**Figure 1:**
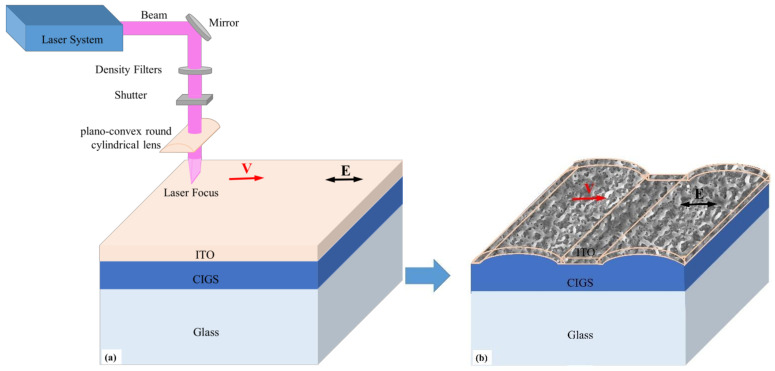
Schematic diagram of processing: (**a**) the laser irradiates the CIGS/ITO bilayer films perpendicular to their surface, and the scanning direction parallel to the laser polarization, (**b**) the fabrication of micro and nano-structures. The black arrow represents the laser polarization.

A CIGS film of 1.5 μm thickness was sputtered onto a series of glass substrates via magnetron sputtering at room temperature (DISCOVERY635, Denton Vacuum, Moorestown, NJ, USA). Then, an additional ITO thin film of 50 nm was sputtered onto the CIGS film surface. Form this, an Nd:YLF femtosecond laser system (Spitfire Ace-120F, Spectra-Physics, Santa Clara, CA, USA) with a pulse regenerative amplification of 800 nm wavelength and a repetition rate of 1 kHz was used. The laser system, which has a maximum power of 5 W, delivered pulses of 120 fs duration from a rectangular beam of 9 μm × 13 mm via a plano-convex round cylindrical lens having a 75 mm focal length. The energy density distribution of the laser beam was Gaussian, and the beam quality factor (M^2^) measured 1.3. The laser pulse energy was adjusted by using a combination of a half-wave plate and a linear polarizer. The sample was fixed onto a motorized xyz stage (OWIS, PS-30, OWIS GmbH, Staufen, Germany) to ensure a precise positioning of the sample. The overlap of the pulses that were irradiated onto the sample surface was tuned via a mechanical shutter and by controlling the scanning speed of the laser. The schematic diagram of the fabrication of the periodic structures is shown in Figure 1. The laser irradiated the CIGS/ITO bilayer films perpendicular to their surface, and the scanning direction was parallel to the laser polarization. The overlapping rate of the laser spot was modulated by the scanning speed. With laser pulses interacting with the bilayer films continuously, control of the ultrafast photon—electron–phonon interactions was facilitated. Therefore, the morphologies of periodic structures can be controlled by appropriate processing parameters. The structure morphology was observed and analyzed via scanning electron microscopy (SEM, SU-8010, Hitachi, Tokyo, Japan), atomic force microscopy (AFM, INNOVA, Bruker, Karlsruhe, Germany), and the compositions were observed by energy dispersive spectrometry (EDS, Hitachi, Tokyo, Japan). Furthermore, optical transmittances of the prepared films were measured by an ultraviolet spectrometer (UV-3600, Shimadzu, Tokyo, Japan).

## 3. Results and Discussion

### 3.1. Field Intensity Analysis and Effect on Surface Structures

**Figure 2:**
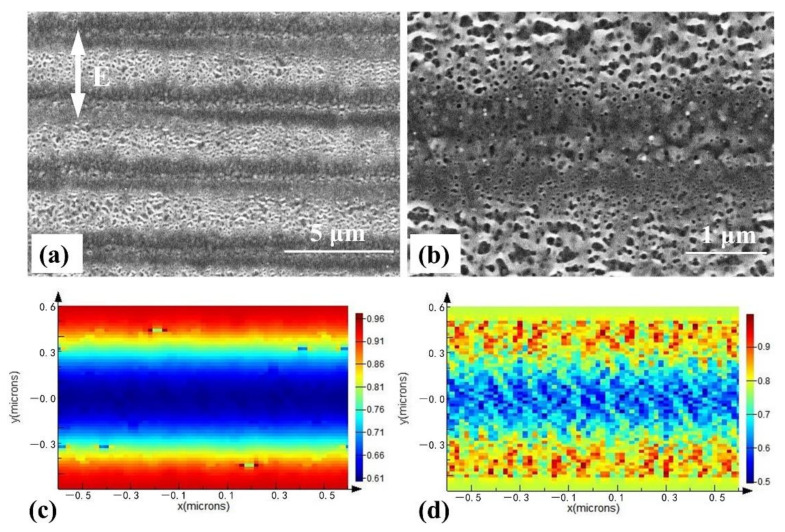
Morphology and field intensity at different layer positions of CIGS/ITO bilayer films: (**a**) Morphology of the surface structures fabricated by using a pulse energy of 0.1 μJ and a scanning speed of 1 mm/s, and (**b**) is partial magnified image of (**a**). (**c**,**d**) are the field intensities at the upper part of CIGS film and the lower part of ITO film, respectively. The white arrow represents the laser polarization.

Compared with single-layer films, bilayer films exhibit a special field intensity distribution under laser irradiation. The morphology and field intensity at different layer positions are shown in Figure 2. As the absorptive layer, CIGS films exhibit low transmittance but a higher thickness than the upper layer of ITO films. The surface of CIGS/ITO bilayer films shows a periodic corrugated structure, which accompanies some porous structures as shown in Figure 2a. The field intensity in the upper part of CIGS film presents obvious periodic distribution (Figure 2c). Field intensity in the lower part of the ITO film showed a fragmented distribution in addition to the periodic corrugated distribution due to optical reflection at the interface and later interference with the CIGS/ITO bilayer films (Figure 2d). The corresponding surface morphology is shown in Figure 2b. Thus, surface structures with a large period accompanied by spatial porous structures can be fabricated under the appropriate conditions.

### 3.2. Fabrication of Different Surface Structures

**Figure 3:**
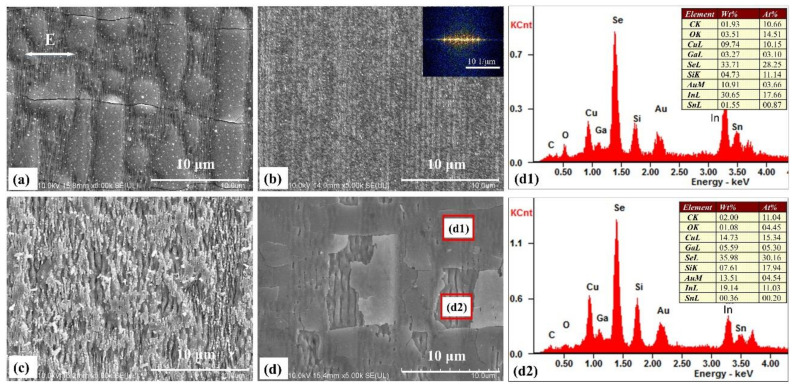
Morphology of the surface structures fabricated by using a laser pulse energy and a scanning speed of: (**a**) 0.1 μJ, 0.01 mm/s, (**b**) 0.1 μJ, 0.1 mm/s, (**c**) 0.2 μJ, 0.01 mm/s, (**d**) 0.2 μJ, 0.05 mm/s, respectively. The corresponding fast Fourier transform of (**b**) is inserted in the upper-right corner. The red rectangle in (**d**) indicates the different positions of remaining film surface and crack, and the corresponding compositions are analyzed in (**d1**,**d2**), respectively. The white arrow represents the laser polarization.

All the morphology of the surface structures can be affected by a variation in laser pulse irradiation (Figure 3). A low pulse energy of 0.1 μJ and a slow scanning speed of 0.01 mm/s were used, and the light was reflected at the interface of the CIGS/ITO bilayer film by exploiting the high transmittance of the ITO thin-film layer. In this fashion, the laser beam impinged on the top surface of the CIGS film. The energy deposited at the interface between the ITO and the CIGS thin films produced a structural pattern onto the lower surface of the ITO thin film and onto the top surface of the CIGS film. Due to the use of low-energy pulses, the ITO film remained intact. However, several deformations caused by the periodic distribution of the light intensity generated an insurgence of cracks into the material (Figure 3a). By increasing the scanning speed to 0.1 mm/s, the spot overlap rate decreased and no significant deformation of the ITO thin film was observed. A large area consisting of structures with a period of 600 nm can be fabricated on the top surface of the ITO films, and the corresponding fast Fourier transform was inserted in the upper-right corner (Figure 3b). By increasing the pulse energy to 0.2 μJ, the top layer of the ITO film appeared to be seriously damaged. Moreover, the periodic surface structures were only partially conserved at a lower scanning speed of 0.01 mm/s (Figure 3c). When the scanning speed increased to 0.5 mm/s, several deformations appeared on the ITO film, leading to a higher density of cracks; even some fragments of the ITO may have fallen off. The bottom layer of the CIGS film was exposed (Figure 3d). The compositions in the deformed film and the crack, which are indicated by the red rectangle in Figure 3d, are shown in Figure 3d1,d2, respectively. Compare with the change of the weight ratio and atomic percentage, there is almost no residual ITO at the crack. Moreover, high-spatial-frequency periodic surface structures (HSFL) are formed on the ITO thin-film surface when a higher spot overlap rate with a small scanning speed of 0.01 mm/s is used (Figure 3a,c). These features disappear when the overlap decreases with the scanning speed increases (Figure 3b,d).

**Figure 4:**
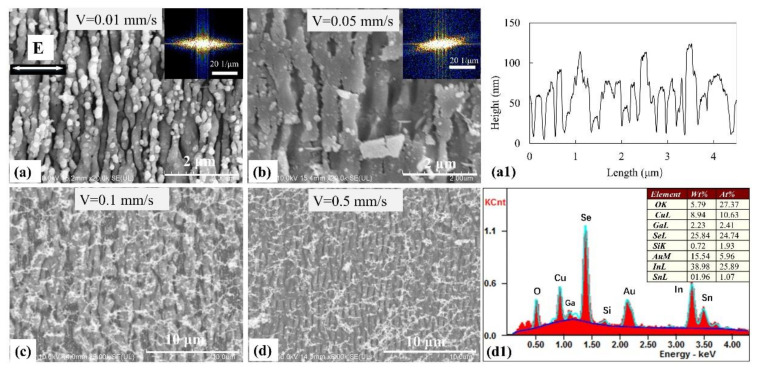
Morphology of the surface structures fabricated by using a pulse energy of 0.15 μJ and a scanning speed of (**a**) 0.01 mm/s, (**b**) 0.05 mm/s, (**c**) 0.1 mm/s, (**d**) 0.5 mm/s. The corresponding fast Fourier transform of (**a**,**b**) are inserted in the upper-right corner, respectively. (**a1**) is the topography profile of (**a**), and (**d1**) indicates the compositions of (**d**). The white arrow represents the laser polarization.

In order to investigate the influence of different parameters on the surface structures of the CIGS/ITO bilayer film, different morphologies were fabricated using a laser pulse energy of 0.15 μJ and a variable scanning speed. The results are shown in Figure 4. At a scanning speed of 0.01 mm/s, the ITO thin film appeared to be significantly damaged. The lower CIGS film layer was exposed and low-spatial-frequency periodic surface structures (LSFL) with a period of 500 nm were fabricated. The high-spatial-frequency periodic surface structures with a period of 200 nm of the residual ITO film remained on the surface of the CIGS film (Figure 4a) and the corresponding fast Fourier transform was inserted in the upper-right corner. The topography profile in Figure 4a1 clearly shows the two periods of structures. For a scanning speed of 0.05 mm/s, damage can be induced on the ITO film and the residual spatial structures showed a low-frequency period. Surface structures with a period of 700 nm were also fabricated onto the lower layer of the CIGS film (Figure 4b) and the corresponding fast Fourier transform is inserted in the upper-right corner. After increasing the scanning speed to 0.1 mm/s, damage to the ITO films decreased (Figure 4c). The sample remained completely intact for a scanning speed of 0.5 mm/s (Figure 4d). The compositions are indicated in Figure 4d1. Flocculent deposition accompanied the fabrication of the periodic structures on the surface of the ITO films.

**Figure 5:**
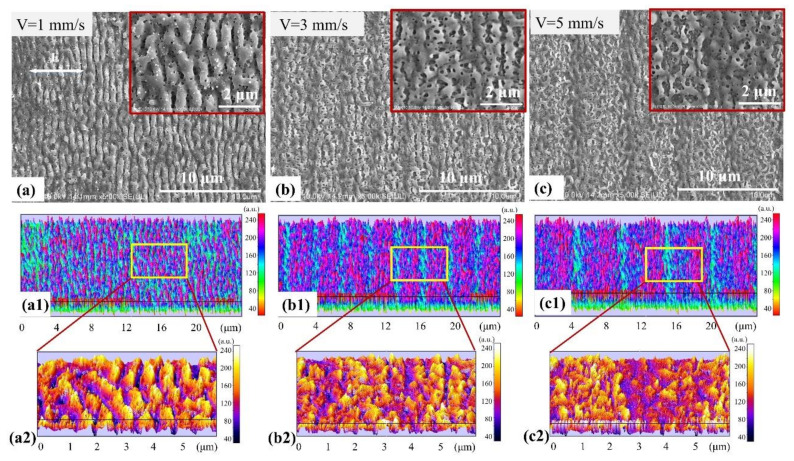
Morphology of the surface structures fabricated by using a pulse energy of 0.15 μJ and a scanning speed of: (**a**) 1 mm/s, (**b**) 3 mm/s, and (**c**) 5 mm/s. The corresponding magnified images are inserted of red rectangles in each upper-right corner. (**a1**,**b1**,**c1**) and (**a2**,**b2**,**c2**) are the corresponding interactive surface plots and detailed morphologies and sizes of (**a**–**c**), respectively. The white arrow represents the laser polarization.

The flocculent deposition decreased upon increasing the scanning speed, and disappears at a speed of 1 mm/s. Uniform periodic ripples were fabricated and the ITO thin film remained intact. The ripples were not perfectly straight and the period measured about 700 nm (Figure 5). Several small porous structures were produced inside the periodic ones (Figure 5a) and its magnified image is in the red rectangle in the upper-right corner. The corresponding interactive surface plots and details are shown in Figure 5a1,a2, respectively. For a scanning speed of 3 mm/s, more porous structures with a larger period of ripples were obtained, which may have reduced the reflectivity of the bilayer films. The period measured about 3 μm. No deposition onto the surface of the ITO thin film was observed; however, the number of nanoscale porous structures increased and became looser, and a larger number of multilayers appeared (Figure 5b). Its magnified image is in red rectangle in the upper-right corner. The corresponding interactive surface plots and details are shown in Figure 5b1,b2, respectively. When the scanning speed reached 5 mm/s, the ITO thin film remained intact and the period of the surface structures was about 5 μm. However, the number of porous structures decreased (Figure 5c) and its magnified image is in red rectangle in the upper-right corner. The corresponding interactive surface plots and details are shown in Figure 5c1,c2, respectively. Thus, the structures fabricated using a scanning speed of 3 mm/s may exhibit such a low reflectivity value due to the light-trapping effect generated by the presence of the porous structures.

### 3.3. Optical Performance Analysis and Optimization

**Figure 6:**
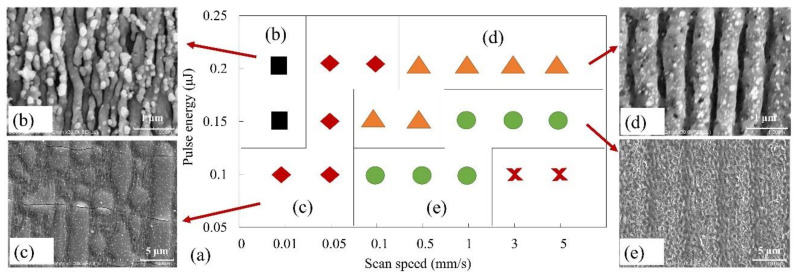
(**a**) Summary of selected scanning speed (0.01–5 mm/s) and laser pulse energy (0.1–0.2 μJ) conditions, where different surface structures were induced on CIGS/ITO bilayer films. The red cross indicates that there is no structure fabricated on the planar surface. (**b**–**e**) are the representative morphologies of each part: (**b**) LSFL + HSFL, (**c**) Deformations or crack formations in the upper ITO film, (**d**) Surface with ripple structures, (**e**) Surface with ripples accompanied by porous structures.

Figure 6a shows the results of selected scanning speed (0.01–5 mm/s) and laser pulse energy (0.1–0.2 μJ) conditions, where different surface structures were induced on CIGS/ITO bilayer films. Figure 6b–e shows the representative morphologies of each part: (b) shows the low-spatial-frequency periodic surface structures accompanied by some high-spatial-frequency periodic surface structures, (c) shows deformations or crack formations in upper ITO film, (d) shows the surface with ripple structures, and (e) shows the surface with ripples accompanied by porous structures. Figure 6f shows the planar surface without any structures.

**Figure 7:**
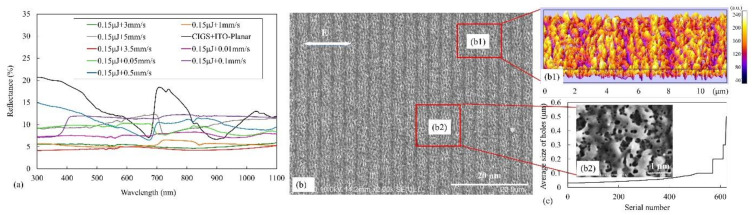
(**a**) Reflectivity of bilayer films with different surface structures, (**b**) Morphology of optimal result fabricated by using a scanning speed of 3.5 mm/s and a laser pulse energy of 0.15 μJ. (**b1**) is the corresponding interactive surface plots and details. (**b2**) is the magnified image of the red rectangle in (**b**), and (**c**) is the corresponding analysis of the holes in the porous structure. The white arrow represents the laser polarization.

Figure 7a shows the reflectivity of different surface structures which are located onto the bilayer films surface. The bilayer thin films with untreated planar surface exhibited a higher reflectivity, except in a wavelength near 680 and 900 nm. The reflectivity decreased near these two wavelengths because of the resonance effect. Moreover, the reflectivity of the processed films decreased and became more uniform in the 300–1100 nm wavelength range. As was seen, these surface structures showed better optical performance of the sample compared to planar surfaces. The corresponding surface characterized by the ripples had a lower reflectivity than the untreated planar surface, which showed antireflection in many areas. Moreover, the ripples accompanied by porous structures further reduced the reflectivity of the bilayer films due to the light trapping effect of the porous structures. Results with good properties were obtained using a pulse energy of 0.15 μJ and a scanning speed in the range of 1–5 mm/s because reflectivity increased considerably at a speed of 5 mm/s. The period of the microstructures was great, whereas the porous structures become weak.

The optimal areas with ripples accompanied by porous structures were successfully fabricated using a scanning speed of 3.5 mm/s and a pulse energy of 0.15 μJ (Figure 7b). The corresponding interactive surface plots and details are shown in Figure 7b1. Their period measured about 3.5 μm. Both the distribution and the size of nanoscale porous structures became more uniform. The magnified image of such an optimal sample corresponds to Figure 7b2. The holes were counted and analyzed (Figure 7c). As can be seen, the average size of most holes was below 0.1 μm and only some of the holes are submicron. These uniform porous structures can greatly decrease the reflectivity, which is lower than 5%. These results show that large periodic areas characterized by ripples accompany by porous structures, which exhibit a high optical performance and a high-efficiency, can be fabricated.

## 4. Conclusions

This study proposed a novel method for processing CIGS/ITO bilayer films by using a rectangular spot irradiated by a femtosecond laser. Bilayer films exhibited different optical characteristics than found in a single-film layer. Field intensities at different layer positions of the CIGS/ITO bilayer films were very different, which showed an obvious periodic distribution in the upper part of the CIGS film and a fragment distribution besides a periodic distribution in the lower part of the ITO film. The optics were reflected at the interface and later interfered with the CIGS/ITO bilayer films. Different structures were successfully fabricated by adjusting a set of parameters. High-frequency laser-induced periodic spatial structures were fabricated onto ITO thin film with a higher spot overlap rate and with a low scanning speed of 0.01 mm/s and a pulse energy of 0.1 μJ. By fixing the pulse energy at 0.15 μJ and varying the scanning speed in the 0.1–1 mm/s range, ripples were obtained. For a larger scanning speed in the 3–5 mm/s range, large periodic structures accompanied by spatial porous structures were were obtained at a scanning speed of 3.5 mm/s and a pulse energy of 0.15 μJ, which had a reflectivity lower than 5%. Large areas characterized by ripples and porous structures with a high optical performance and efficiency were successfully fabricated, which can improve the field of application of thin-film-based solar cells.

## Data Availability

The data presented in this study are available on request from the corresponding author.
